# Metabolic Responses to High‐Fat Feeding and Chronic Psychological Stress Combination

**DOI:** 10.1002/edm2.487

**Published:** 2024-06-12

**Authors:** Marzieh Nemati, Fatemeh Rostamkhani, Roxana Karbaschi, Homeira Zardooz

**Affiliations:** ^1^ Department of Physiology, School of Medicine Shahid Beheshti University of Medical Sciences Tehran Iran; ^2^ Endocrinology and Metabolism Research Center Shiraz University of Medical Science Shiraz Iran; ^3^ Department of Biology, College of Basic Sciences, Yadegar‐e‐Imam Khomeini (RAH) Branch Islamic Azad University Tehran Iran; ^4^ Faculty of Nursing and Midwifery Shahid Beheshti University of Medical Sciences Tehran Iran; ^5^ Neurophysiology Research Center Shahid Beheshti University of Medical Sciences Tehran Iran

**Keywords:** chronic stress, corticosterone, high‐fat diet, insulin resistance

## Abstract

**Introduction:**

High‐fat diet (HFD) consumption and being exposed to daily psychological stress, common environmental factors in modern lifestyle, play an important role on metabolic disorders such as glucose homeostasis impairment. The aim of this study was to investigate the effects of high‐fat diet (HFD) and psychological stress combination on metabolic response to chronic psychological stress in male rats.

**Method:**

Male Wistar rats were divided into HFD, and normal diet (ND) groups and then into stress and nonstress subgroups. The diets were applied for 5 weeks, and psychological stress was induced for 7 consecutive days. Then, blood samples were taken to measure glucose, insulin, free fatty acids (FFA), and leptin and corticosterone concentrations. Subsequently, glucose‐stimulated insulin release from pancreatic isolated islets was assessed.

**Results:**

HFD did not significantly change fasting plasma glucose, insulin and corticosterone levels, whereas increased plasma leptin (7.05 ± 0.33) and FFA (*p* < 0.01) levels and impaired glucose tolerance. Additionally, HFD and stress combination induced more profound glucose intolerance associated with increased plasma corticosterone (*p* < 0.01) and leptin (8.63 ± 0.38) levels. However, insulin secretion from isolated islets did not change in the presence of high‐fat diet and/or stress.

**Conclusion:**

HFD should be considered as an intensified factor of metabolic impairments caused by chronic psychological stress.

## Graphical abstract

High‐fat diet (HFD) consumption and being exposed to daily psychological stress, common environmental factors in modern lifestyle, play an important role in metabolic disorders such as glucose homeostasis impairment. The aim of this study was to investigate the effects of HFD and psychological stress combination on metabolic response to chronic psychological stress in male rats.

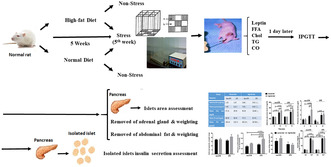

## 1 Introduction

Impaired insulin action and secretion is one of the most important causes of glucose metabolism‐related disorders [1, 2, 3]. It happens because of multiple environmental factors including the HFD consumption and exposure to stress [4, 5]. The results of in vitro [6, 7] and in vivo [8, 9] investigations indicated that long‐term elevation of free fatty acid (FFA) inhibits glucose‐stimulated insulin secretion (GSIS) of islets. Also, HFD led to an increase in plasma leptin level, followed by an increase in food consumption, body weight and insulin resistance [10]. Leptin reduced pancreatic beta cell insulin secretion [11, 12] via activating beta cells' ATP‐sensitive potassium channels and hyperpolarisation [13, 14]. Also, leptin through its antagonistic effect on the GLP‐1 [15] indirectly affects the amount of insulin secretion and possibly number of islets. Thus, leptin by reducing the release of insulin can prevent fat production and insulin resistance induction in fat cells. However, some studies showed that most HFDs caused the leptin resistance [15, 16, 17, 18] and consequently glucose homeostasis impairment. High‐fat foods with a high percentage of saturated fat elevated plasma leptin level, which is associated with reduced insulin sensitivity, while replacing saturated fat with unsaturated fat decreased body weight and increased insulin sensitivity [19, 20, 21]. Stress is one of the most important stimulatory factors of glucocorticoids secretion that by triggering corticosterone and/or epinephrine release increases gluconeogenesis, glycogenolysis, inhibits insulin secretion, induces insulin resistance and impairs glucose homeostasis [22, 23, 24]. It should be noted that a HFD increases the HPA axis response to stress [25, 26]; however, a decrease in the HPA axis response to stress in the presence of HFD was shown in some other studies [27, 28]. Stress have different effects on plasma leptin level, depending on the modality and duration of stress. During exposure to stress, sympatho‐adrenal axis stimulation decreases plasma leptin level, whereas the HPA axis stimulation increases plasma corticosterone and leptin levels [29]. In animals that consumed HFD and exposed to chronic stress, plasma glucose and insulin were higher than in animals in the presence of HFD or chronic stress alone [5]. Therefore, it seems that HFD in combination with stress plays a crucial role in the induction and development of glucose‐related metabolic disorders. Our previous research showed that a HFD containing cow intra‐abdominal fat did not have a negative effect on the metabolic responses induced by the acute exposure to psychological stress [30]. Consequently, in the present study, we aimed to clarify whether this kind of HFD (67.66% of the energy from cow intra‐abdominal fat and 54% of it is unsaturated fatty acid), which is available and used mostly for frying foods [30, 31, 32], affects the metabolic response to chronic exposure to psychological stress (a prevalent stressor in today's societies). Undoubtedly, results of this investigation can be helpful in the management of clinical metabolic responses to chronic stress and would open new window regarding to this issue. In this study, the parameters related to glucose metabolism in animal model of a HFD exposing to chronic psychological stress were evaluated.

## 2 Materials and Methods

### 2.1 Animals and Ethical Statement

Twenty‐eight male Wistar rats (10–12 weeks) weighing 170 ± 5 g were used. In each cage, two animals were kept under controlled temperature (22 ± 2°C) and constant light–dark cycle (light from 7:00 AM to 7:00 PM) with ad libitum food and water.

### 2.2 Study Design

Animals were randomly divided into two groups of normal diet (ND) (standard pellet, 4.75% energy as fat, pars production and distribution of animal feed company, Iran) and HFD (cow intra‐abdominal fat mixed with standard pellets, 66.67% energy as fat). The intra‐abdomina fat of cows exhibits a fatty acids profile consisting of 54.21% saturated fatty acids and 44.89% unsaturated fatty acids [31]. After 30 days, animals of each group were randomly allocated into nonstress (non‐STR) and stress (STR) subgroups, whereas they continued their respective diets. At the end of the experiment, body weight, food and energy intake of animals were measured, blood samples were taken to measure plasma parameters levels, intraperitoneal glucose tolerance test (IPGTT) was performed, and pancreases were removed to assess isolated islets' insulin secretion.

### 2.3 Stress Induction

Communication box was used to induce psychological stress. It (BorjeSanat, Tehran, Iran) was consisted of nine chambers (16 × 16 × 50 cm). The animals in the chambers had visual, auditory and olfactory communication with each other. The floor of five chambers was made of stainless steel grids in which the animals of the chambers were put under electrical shock. The remaining four chambers' floors were covered by plastic plates, and the animals received cues such as visual, auditory and olfactory sensations from the neighbouring animals who exposed to electric foot shock, so they were under psychological stress. Stress exposure lasted for 1 h between 11:00 AM and 13:00 PM for 7 consecutive days. It is noteworthy that the control non‐STR groups were kept in the box without receiving any stress for the same time [33].

### 2.4 Blood Sampling

Immediately after removing animals from the communication box (8–8:30 AM), they were anaesthetised by isoflurane (Nicholas Primal, London, UK) [34] and blood samples were taken using retro‐orbital puncture method and collected in Eppendorf tubes containing 5 μL heparin (Caspian Tamin, Rasht, Iran) (5000 IU/mL) per 1 mL blood; tubes were centrifuged at 3000 rpm for 5 min; and plasma was kept at 80°C to assess leptin, cholesterol, triglycerides, FFAs and corticosterone concentrations.

### 2.5 Hormones and Biochemical Assessment

For determining plasma corticosterone and leptin concentrations, corticosterone enzyme‐linked immunosorbent assay (ELISA) kit (DRG, Germany) and rat leptin ELISA kit (Mercodia, Sweden) were used, respectively. Plasma triglyceride (TG) and cholesterol (Chol) levels were measured by colorimetric method (Pars Azmoon, Iran). Plasma FFA levels were determined by a rat FFA colorimetric kit (Nanjing Jiancheng Bioengineering Institute, Nanjing, China).

### 2.6 Intraperitoneal Glucose Tolerance Test (IPGTT)

IPGTT was performed 1 day after the last stress exposure after 16 h fasting. Blood sample was taken in fasting status and also 15 min after the IP injection of glucose (2 g/kg of glucose 20% solution) in order to measure plasma glucose and insulin concentrations [32]. To assess plasma insulin, rat insulin ELISA kit (Mercodia, Sweden) was used. Moreover, plasma glucose concentration was determined using the glucose oxidase method (Pars Azmoon, Iran).

### 2.7 Adrenal and Abdominal Fat Weight

After performing IPGTT, under anaesthesia the animals of all experimental groups were decapitated, their adrenal glands and left‐side abdominal fat were removed and weighted [32].

### 2.8 Assessment of the Langerhans Islet Area

The pancreases of three rats of each group were totally removed and immersed in 10% formalin buffer for 24 h, then embedded in paraffin and cut into 5‐μm sections frontally and stained with haematoxylin–eosin [32]. A serial section was provided from each pancreas, and 10 sections from the middle, intestinal and spleen areas were randomly examined by the MOTIC software (Nikon, Japan, 2001).

### 2.9 Islet Isolation Procedure

The abdomen of the remaining four rats, who were decapitated, was opened, and the entrance of common bile duct to duodenum was clamped; then, duct was cannulated with polyethylene catheter, and pancreas was perfused with 10 mL of cold Hanks’ buffer containing collagenase P (Roche, Cat. No. 11213865001; Mannheim, Germany, 0.45 mg/mL). The inflated pancreas was removed and cleaned from nonpancreatic tissue and then incubated for 17 min at 37°C in water bath. After shaking, the digested pancreases were washed with cold Hanks’ solution. The supernatant was removed, and precipitated islets were transferred to petridish to be handpicked under stereomicroscope [35].

### 2.10 Assessment of Insulin Secretion From the Isolated Islets

To assess GSIS, 10 handpicked isolated islets were incubated with different glucose concentrations (5.6 and 16.7 mM) for 90 min; then, the supernatant was removed, and insulin secretion from isolated islets were measured using rat insulin ELISA kit (Mercodia, Sweden) [32].

### 2.11 Statistical Analysis

The results were analysed by SPSS, Version 9.0, program package (Nie NH, Hull CH, Bent DH, Stanford, CA). All data are expressed as mean ± SEM. A three‐factor mixed model analysis of variance (ANOVA) (time was considered as a repeated factor, and diet and stress were considered as independent factors) and two‐ and three‐way ANOVAs (considering diet and stress; and diet, stress and glucose concentrations as independent factors, respectively) were performed and followed by Tukey's post hoc test. *p*‐values below 0.05 were considered to be statistically significant.

## 3 Results

### 3.1 Body Weight

There was no significant difference in body weight of animals who received a ND compared with those who received HFD. Moreover, psychological stress did not change the animals' body weight compared with the non‐STR condition (Table 1).

**TABLE 1:** The effect of HFD and/or chronic psychological stress on body weight, food and energy intake, abdominal fat and plasma leptin, cholesterol, triglyceride and free fatty acids concentrations.

Parameter	Groups			
	ND		HFD	
	Non‐STR	STR	Non‐STR	STR
Body weight (g)	222.33 ± 4.41	245.14 ± 5.32	234.30 ± 8.79	239.76 ± 4.83
Food intake (g/rat/24 h)	15.44 ± 0.59	16.23 ± 0.64	10.00 ± 0.26^a^ ^,^ ****	10.46 ± 0.15^a^ ^,^ **** ^,^ ^c^ ^,^ ****
Energy intake (kcal/rat/24 h)	58.68 ± 2.41	61.67 ± 2.45	61.00 ± 1.59	63.79 ± 0.90
Abdominal fat weight (g)	1.28 ± 0.10	2.10 ± 0.14	2.88 ± 0.06^a^ ^,^ ***	3.91 ± 0.37^a^ ^,^ **** ^,^ ^b^ ^,^ * ^,^ ^c^ ^,^ ****
Plasma leptin level (ng/mL)	4.59 ± 0.33	6.22 ± 0.39^a^ ^,^ *	7.05 ± 0.33^a^ ^,^ ***	8.63 ± 0.38^a^ ^,^ **** ^,^ ^b^ ^,^ * ^,^ ^c^ ^,^ ^****^
Plasma cholesterol level (mg/dL)	47.14 ± 4.54	39.43 ± 2.01	50.29 ± 4.48	63.86 ± 3.80^a^ ^,^ * ^,^ ^c^ ^,^ ***
Plasma triglyceride level (mg/dL)	69.00 ± 2.96	66.43 ± 1.51	75.71 ± 4.60	81.43 ± 6.61^c^ ^,^ *
Plasma free fatty acid level (μmol/L)	193.14 ± 9.97	215.71 ± 4.81	302.14 ± 30.55^a^ ^,^ **	287.86 ± 17.52^a^ ^,^ ** ^,^ ^c^ ^,^ *

*Note*: Values are the mean ± SEM of 6–7 rats.

Abbreviations: HFD, high‐fat diet; ND, normal diet; STR, stress.

^a^ Represents significant difference versus non‐STR group of ND rats.

^b^ Represents significant difference versus non‐STR group of HFD rats.

^c^ Represents significant difference versus STR group of ND rats.

* *p* < 0.05.

** *p* < 0.01.

*** *p* < 0.001.

**** *p* < 0.0001.

### 3.2 Food and Energy Intake

As shown in Table 1, there was no significant difference in food intake between STR and non‐STR of the ND groups. HFD with and without stress caused a significant decrease in food intake compared with the ND groups (*p* < 0.0001). However, there were no significant differences in energy intake among four experimental groups (Table 1).

### 3.3 Intra‐Abdominal Fat Weight and Plasma Leptin Concentration

The intra‐abdominal fat weight was significantly increased in the HFD groups both in the presence (*p* < 0.0001) and in the absence (*p* < 0.001) of stress compared with the ND groups. Moreover, it was markedly higher in the STR group of HFD rats than the non‐STR group of the same diet (*p* < 0.05) (Table 1).

HFD significantly increased plasma leptin concentration in both STR (*p* < 0.0001) and non‐STR (*p* < 0.001) conditions compared with the ND groups. Moreover, leptin plasma level in both ND and HFDs was significantly higher in the STR groups than in the non‐STR groups (*p* < 0.05) (Table 1).

### 3.4 Plasma Cholesterol, Triglyceride and Free Fatty Acids Concentrations

Although HFD or STR alone did not affect the plasma TG concentration; HFD‐STR could significantly increase this plasma factor compared with the same group with ND (*p* < 0.05). Plasma Chol level was also increased in animals that consumed HFD in the presence of the STR compared with the animals of the non‐STR (*p* < 0.05) and STR groups (*p* < 0.001) with ND (Table 1).

Plasma FFA concentrations were higher in the HFD groups in the absence or presence of stress than in the non‐STR‐ND group (*p* < 0.01) (Table 1). Moreover, in the STR group of HFD, the levels of FFA were higher than those of the STR group of ND (*p* < 0.05) (Table 1).

### 3.5 Plasma Corticosterone Concentrations and Adrenal Gland Weight

**FIGURE 1:**
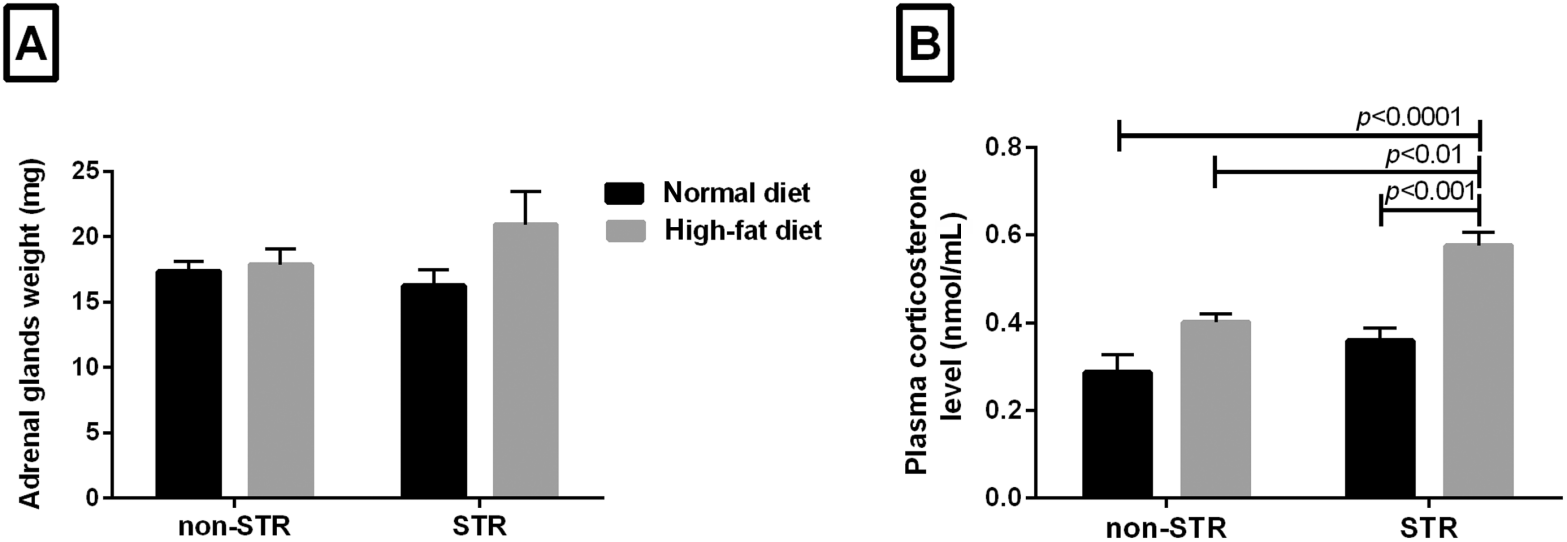
Effect of high‐fat diet and/or chronic psychological stress on adrenal glands weight (A) and plasma corticosterone (B). Each column represents mean ± SEM of seven rats. HFD, high‐fat diet; ND, normal diet; non‐STR, non stress; STR, stress.

As shown in Figure 1A, there were no significant differences in adrenal gland weight among four experimental groups.

HFD alone had no significant effect on plasma corticosterone concentration; however, HFD and STR combination significantly increased corticosterone concentration compared with the non‐STR group of the same diet (*p* < 0.01), STR (*p* < 0.001) and non‐STR (*p* < 0.0001) groups with ND (Figure 1B).

### 3.6 Plasma Glucose and Insulin Concentrations Before and After IPGTT

**FIGURE 2:**
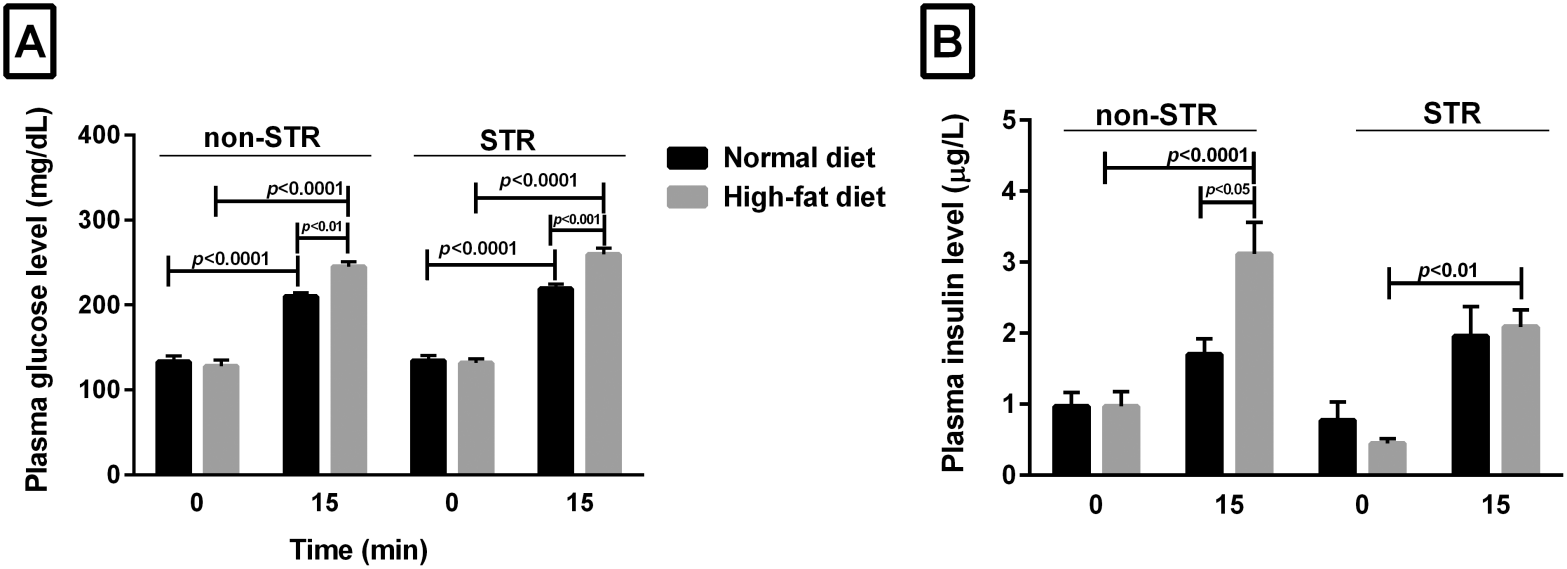
Effect of HFD and/or chronic psychological stress on plasma glucose (A) and insulin (B) concentrations in fasting state (0 min) and 15 min after performing IPGTT. Each column represents mean ± SEM of seven rats. HFD, high‐fat diet; ND, normal diet; non‐STR, non stress; STR, stress.

Fifteen minutes after glucose tolerance test in both the ND and HFD groups, plasma glucose concentration was significantly higher than in the fasting state (zero time) (*p* < 0.0001) (Figure 2A); however, STR and/or HFD had no significant effect on plasma glucose concentration in the fasting state (zero time) (Figure 2A). On the contrary, in animals consuming HFD, plasma glucose concentration, 15 min after GTT, was significantly higher in the stressed (*p* < 0.001) and non‐STR (*p* < 0.01) groups than the same groups which fed ND (Figure 2A).

Fasting plasma insulin concentration (zero time) was not significantly different between the HFD or ND group in the presence or absence of stress. However, this plasma parameter increased 15 min after glucose tolerance test in all groups compared with their zero time, which was significant only in the non‐STR HFD group (*p* < 0.0001) (Figure 2B). In the non‐STR HFD group at 15 min, the level of insulin was also higher than that in the nonstressed ND group at the same time (*p* < 0.05). In the stressed HFD group, 15 min after glucose tolerance test, a higher plasma insulin level was observed than in the same group at 0 min (*p* < 0.01) (Figure 2B).

### 3.7 Area of the Langerhans Islets and Insulin Secretion From Isolated Islets of Langerhans

**FIGURE 3:**
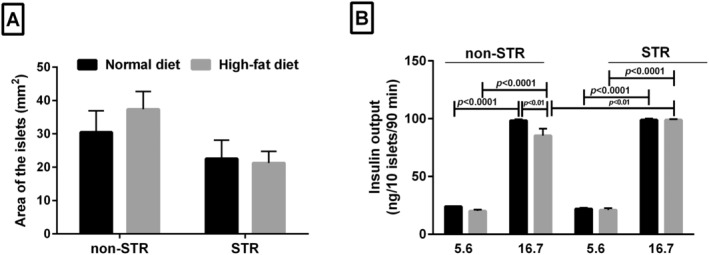
Effect of HFD and/or chronic psychological stress on the area of the islets (A), each column represents mean ± SEM of three rats; and isolated pancreatic islets insulin secretion in response to glucose (B), each column represents mean ± SEM of four rats (5 groups of 10 islets each/glucose concentration/rat). HFD, high‐fat diet; ND, normal diet; non‐STR, non stress; STR, stress.

None of the HFD and/or STR had significant effect on the area of the Langerhans islets (Figure 3A).

In the presence of 5.6 mM glucose concentration, HFD or STR, either alone or in combination, had no significant effect on the level of islets insulin secretion, whereas HFD alone significantly reduced insulin secretion from islets (*p* < 0.01) in the presence of 16.7 mM glucose concentration (Figure 3B). However, insulin secretion from the islets was significantly higher in combined HFD and STR in 16.7 mM glucose concentration than in the non‐STR group (*p* < 0.01). Insulin secretion from isolated islets in the 16.7 mM glucose concentration was remarkably higher than insulin release in 5.6 mM glucose concentration in all groups (*p* < 0.0001) (Figure 3B).

## 4 Discussion

The results revealed that HFD enhanced intra‐abdominal fat weight and plasma concentrations of leptin and fatty acids, hence reducing food intake. Also, HFD outperformed the ND group by preventing the rise in energy intake and body weight. In line with this finding, a 6‐week HFD containing 40% lipids did not cause any significant changes in body weight and energy intake. Yet, the HFD group had significantly higher levels of epididymis and visceral fat weight than the control group [36]. In C57BL/6J male mice, a 3‐month HFD (60% fat) increased epididymis and visceral fat weight, while significantly reducing food intake compared with the ND group. In contrast to our results, a study has found the higher body weight and energy intake in the HFD mice than in the ND group [37]. Fifteen‐week HFD (80% unsaturated fat) in male rats either in the presence or in the absence of stress (1 h/day, for 5 days) significantly decreased food consumption. These animal experiments have shown that HFD had different effects depending on stress levels. Without stress, HFD boosted energy intake and increased the weight of visceral, epididymis and peritoneal fat, resulting in a significant gain in body weight. With stress; however, HFD did not alter these parameters. In rodents, unlike humans, a decrease in food consumption has been observed following stress, which is associated with a decrease in body weight or lack of weight gain [38, 39]. STR did not change food intake, energy intake and body weight, which perhaps confirms that the STR used in this study was not intense enough to affect these parameters. In this regard, it has been shown that the relationship between stress and reduction of food consumption and body weight is a reliable marker in evaluating the intensity and severity of stress model [40].

Stress in the HFD group caused a significant enhancement in plasma corticosterone concentration, which was associated with a proportional increase in intra‐abdominal fat weight and plasma leptin concentration. A previous study has shown that leptin levels in the blood were higher in both the HFD group and the stress group than in the corresponding groups with ND [40]. Hence, an increase in intra‐abdominal fat and plasma leptin level is one of the signs of the effect of elevated corticosterone concentration following chronic stress [32, 41]. However, weight stability despite an increase in fat can be due to the role of corticosterone in breaking proteins down [42].

HFD enhanced plasma FFA concentration, but it did not change the concentrations of Chol and TG. In agreement with our results, 4‐ to 5‐week HFD (50% of calories from fat) in Wistar rats and a 6‐week HFD (60% of energy from fat) in healthy female cats increased plasma FFA concentration [43, 44], without causing significant changes in plasma concentrations of Chol and TG [44]. In contrast, although a 3‐week HFD (60% of energy from fat) in male rats did not change FA level in the fasting condition, it did so in a nonfasting condition. These animals had a lower serum concentration of TG than in the control group [45]. Stress did not affect plasma lipid parameters in the ND group. In line with our finding, after 30 days of ND in mice, a chronic social defeat stress did not cause any significant changes in plasma Chol and non‐HDL‐C concentrations [46]. Previous studies have shown that lipid parameters increased in response to forced immobilisation stress in rabbits [47], and different degrees of exam stress [48], as well as acute and chronic emotional arousal stress in human [49]. Responses to stress depend on many factors including intensity, frequency, duration, type of stressor and activity of HPA axis and sympathoadrenal axis (responsible systems responding to stress) [40]. The findings indicated that STR‐induced elevation of plasma Chol and TG concentrations was observed exclusively in HFD. It suggests that HFD increases the susceptibility to lipid profile alterations in response to stress. In sum, given the constancy of fat profile of these animals, it seems reasonable not to find any significant changes in corticosterone concentration following stress in the ND group. Stress may have activated the HPA axis and the sympathetic nervous system (not measured in this study) in the HFD group, leading to lipolysis and increased FAs in the blood. This may have resulted in higher TG and total Chol levels [20]. In addition, it has shown that HFD by itself increases plasma FFA [43], and/or changes in FAs balance [44], hence inducing inflammation [50, 51]. The sympathetic nervous system can be activated by inflammation [52] leading to the release of corticosterone [53]. Thus, it is plausible to assume that the same mechanism is responsible for the elevation of plasma corticosterone levels in response to chronic stress in the HFD group [47]. In other words, HFD may have sensitised animals to stress, inducing adverse effects of chronic psychological stress due to lipid profile changes.

The weight of adrenal gland did not show any significant changes in any of the groups. Previous studies have reported contradictory results; for instance, Harris et al. showed that restraint stress in male rats in the presence and absence of a HFD did not cause a significant change in the adrenal weight [54], whereas Pitts & Bull have reported that a significant increase was observed in adrenal gland weight in the presence and absence of exercise stress in HFD in male Carworth Farms Elias (CFE) rats. It has been suggested that the increase in adrenal weight of HFD rats is probably due to an increase in the activity of HPA axis [55].

HFD or HFD‐STR impaired glucose tolerance. Insulin resistance was found in the HFD group with or without stress. Previous studies have found that peripheral insulin resistance can be due to an increase in the concentration of plasma FAs, reducing fat oxidation capacity and increasing lipid accumulation in skeleton muscle cells, resulting in insulin signalling inhibition [56, 57, 58]. The involvement of leptin in insulin resistance (62) and a significant correlation between leptin and serum lipid profile [59] have been previously observed so that children with markedly enhanced leptin concentration had higher plasma TG, insulin concentrations and insulin resistance than those with low leptin levels [60]. An investigation has found that a decrease in LDL receptor expression and the subsequent increase in plasma cholesterol led to insulin resistance. Hence, the inhibition of cholesterol synthesis can improve insulin action [61].

It has been found that plasma cholesterol elevation due to HFD and stress may help to reduce plasma insulin concentration observed 15 min after IPGTT performance in comparison with non‐STR condition. Wada M. et al. found that an enhancement in the total Chol level can be related to the decrease in insulin production capacity. Thus, total Chol reduction is essential to prevent diabetes in obese people [62]. The accumulation of TG in tissues such as muscle and its plasma level are linked to insulin resistance [63]. Our findings showed an increase in plasma TG only in the HFD‐STR group. However, we did not measure hepatic TG production and liver and muscle TG level, which is a limitation of this study and requires further investigation.

The area of isolated islets did not significantly change in any of the groups. Contrary to our results, 1‐year HFD in mice increased area of islets [64]. In addition, in people with Type 2 diabetes, size of the islets in obese diabetic people was greater than those in lean diabetic people [65]. Increased islet area appears to be an adaptive response to obesity [64]. As the HFD consumption duration in the current study was not long enough to induce obesity, no changes in the area of the islets were anticipated.

The HFD group had a significant decrease in insulin secretion from isolated islets in response to high glucose concentration, which is contrary to the results of the in vivo study. A possible explanation is that insulin‐degrading enzyme (IDE) in the liver breaks down secreted insulin. HFD may have lowered the liver's ability to clear insulin, and thus, despite the expected decrease in insulin secretion due to HFD, a slight increase in plasma insulin level was seen after GTT. Previous studies have shown that different types of FAs reduce the binding of insulin to IDE and the breakdown of insulin by this enzyme in the hepatocytes [66]. The interesting note is that the amount of insulin secretion from the pancreatic isolated islets in the HFD group in the presence of stress did not show a significant difference with the corresponding ND group, which is consistent with the results of the in vivo study (GTT). In other words, HFD in interaction with stress did not have a significant effect on the islets' ability to secrete insulin. The increased sympathetic nervous system activity [67] stimulates the pancreatic islets insulin secretion with elevated corticosterone level, which in turn inhibits the islets insulin secretion. Thus, it may be responsible for the unchanged insulin output from isolated islets in the HFD‐STR group.

In conclusion, the HFD and HFD‐STR elevated the plasma corticosterone and leptin levels, resulting in glucose intolerance. The results indicate that the HFD containing intra‐abdominal cow fat could potentially make animals more susceptible to the adverse impacts of chronic psychological stress on glucose metabolism. However, the combined effect of the diet and stress did not result in the dysfunction of the isolated islets in terms of insulin secretion.

## Author Contributions

**Marzieh Nemati:** conceptualization (equal), data curation (equal), formal analysis (equal), investigation (equal), methodology (equal), supervision (equal), writing–original draft (equal), writing–review and editing (equal). **Fatemeh Rostamkhani:** conceptualization (equal), formal analysis (equal), supervision (equal), writing–review and editing (equal). **Roxana Karbaschi:** conceptualization (equal), formal analysis (equal), writing–review and editing (equal). **Homeira Zardooz:** conceptualization (equal), formal analysis (equal), methodology (equal), supervision (equal), visualization (equal), writing–review and editing (equal).

## Ethics Statement

All experimental procedures were approved by the Animal Care and Use Committee of Shahid Beheshti University of Medical Sciences (registration no.: 13822).

## Conflicts of Interest

The authors declare no conflicts of interest.

## Data Availability

The datasets used and/or analysed during the current study are available from the corresponding author on reasonable request.
